# Adjusting to the Reign of Webinars: Viewpoint

**DOI:** 10.2196/33861

**Published:** 2021-11-12

**Authors:** Mert Karabacak, Burak Berksu Ozkara, Zeynep Ozcan

**Affiliations:** 1 Cerrahpaşa Faculty of Medicine Istanbul University-Cerrahpaşa Istanbul Turkey

**Keywords:** virtual conference, student-based organization, neuroscience conference, COVID-19, medical education, webinars, web-based education

## Abstract

**Background:**

With the integration of COVID-19 into our lives, the way events are organized has changed. The Cerrahpaşa Neuroscience Days held on May 8-9, 2021, was one of the conferences that was affected. The annual conference of the student-based Cerrahpaşa Neuroscience Society transitioned to the internet for the first time and had the premise of going international.

**Objective:**

With this study, we aim to both discuss how a virtual conference is organized and perceived, and where our conference stands within the literature as a completely student-organized event.

**Methods:**

The conference was planned in accordance with virtual standards and promoted to primarily medical schools. During the execution, there were no major issues. The feedback was collected via a form developed with Google Forms.

**Results:**

Out of 2195 registrations, 299 qualified to receive a certificate. The feedback forms revealed a general satisfaction; the overall quality of the event was rated an average of 4.6 out of 5, and the ratings of various Likert scale–based questions were statistically analyzed. Open-ended questions provided improvement suggestions for future events.

**Conclusions:**

The virtual Cerrahpaşa Neuroscience Days was a success in organization and received positive feedback from the participants. We aim to ground future events on this experience.

## Introduction

The integration of COVID-19 into our lives brought along significant changes in the way events are organized. Even though the format of web-based conferences has always been an option, there has never been a time when it was the only one. Therefore, not many were focused on how to optimize virtual conferences. As of August 2021, however, there are plenty of articles based on the experiences of converting a face-to-face conference to a web-based conference [1-4], as well as reviews [5-7] with suggestions to improve the virtual experience. Cerrahpaşa Neuroscience Days was faced with similar challenges to the conferences discussed in the literature with one major difference: it was a student-organized conference.

Cerrahpaşa Neuroscience Society is a student-based organization that was founded in 2018 within Istanbul University-Cerrahpaşa, Cerrahpaşa Faculty of Medicine, and has held several seminars with distinguished professors. Cerrahpaşa Neuroscience Days is the annual conference of the society where several speakers are invited for lectures and social activities are organized for the participants, who are mostly medical students. The first Cerrahpaşa Neuroscience Days was organized in 2019 and it was quite successful in terms of organization and feedback. The conference had a completely national identity with both its speakers and participants. In 2020, the second conference was planned to be executed on April 4-5. On March 11, the novel coronavirus outbreak was declared a global pandemic, and on the next day, the second Cerrahpaşa Neuroscience Days was cancelled with an announcement made over social media platforms. With the uncertainty of the pandemic, the conference had not transitioned to the internet at that time. After 1 year, the conference could still not be held face to face. Therefore, Cerrahpaşa Neuroscience Days was decided to be held on the internet. The society decided to benefit from this change; thus, the 2021 conference had an entirely different premise: going international.

Our conference analysis, as described above, differs from the majority of literature in that it is completely student-organized. There are, however, examples of student-run organizations in the literature [8].

With the acceleration of innovation and renewal in scientific knowledge, undergraduate students want to get involved in academia as early as possible, so as not to lose track of modern science. Not many organizations are easily accessible, especially to undergraduates. Therefore, they organize their own events where they can meet experts on their fields of interest and possibly obtain insight into how to shape their future. Nevertheless, few studies have focused on the process of organizing a conference for students, by students. In this paper, we cover both the student aspect of organizing a conference and how the virtual experience differs from its face-to-face counterpart by reviewing the literature.

## Planning

The critical aspect of the conference was choosing and inviting professors who would present topics that are intriguing for students. In addition, the topics and institutions needed to be diverse. With this in mind, the society’s organization team contacted several professors via email, some of whom the students had not personally known prior to planning and some of whom had personal connections with the students; out of the 6 professors who agreed to participate in the conference, 5 were the latter.

Even though the logistic requirements were much less for a web-based conference than for face-to-face conferences, our conference required financial support. To meet expenses such as conference website hosting and domains and the premium Zoom membership fee, we applied for sponsorships. Several companies were emailed with a digital booklet containing information on the society, the events, and possible sponsorship types. In the end, the event had 1 sponsor.

The event was announced through social media platforms, such as Instagram, Twitter, Facebook, LinkedIn, and WhatsApp. Those registered to our organization’s newsletter also received an email announcing the conference. Medical students were the target audience; thus, contact with medical faculties was prioritized. Our team looked into social media platforms such as Facebook to find foreign organizations to contact. A list of all Turkish medical schools was used to contact the scientific societies of these institutions. The contacted medical schools were asked to announce the event in their local chat groups and on their social media platforms. In addition to this, we used our personal connections to facilitate the promotion of our conference. We began to promote our conference within our own faculty on April 1, 2021, and ended registration on May 7, 2021, with a total of 2195 registrations for passive participation from several countries.

To register participants, an entirely English website of the Cerrahpaşa Neuroscience Society was built as the conference was international. The participants who wanted to register as listeners were instructed to register for the “passive participation” section. The website also included the program arranged for 3 different time zones and information about the speakers.

To ensure participation for certificates, an attendance form was prepared using Google Forms. This form would be sent during each of the lectures with different links. To obtain a certificate, a participant would be required to fill out at least 4 of the 6 forms. Including verification codes for the forms and sending the aforementioned links at randomly chosen points during the lectures increased the reliability of participation from those who filled out the forms.

We wanted this event to present an opportunity to students who are involved in research and want to gain experience in academic presentations. Therefore, we opened registrations for an oral presentation contest and a poster presentation contest. These registrations were named as “active participation.” Participants with a completed research study were asked to send abstracts of their studies to be evaluated by our scientific jury. The jury consisted of 4th- and 5th-year medical students, who were also board members of the society. Out of 20 abstract submissions for oral presentations, 8 were chosen to participate in the contest. Along with these submissions, 9 submissions were made for poster presentations, of which 6 were chosen. Both oral and poster presentations were judged on the basis of the criteria of the jury: meeting the application standards and relevancy to the conference content. The oral presentations were set to take place during the meeting while the posters were presented in a web-based exhibition on MedAll with prerecorded voice overs.

For participants who did not participate in the presentation contests, we still wanted to provide a platform to challenge their medical knowledge. Thus, we decided to hold a case contest where those who sign up would be assigned medical cases in the form of free-text questions. This contest was prepared using the platform HyperSay. The top 3 contestants would win the prizes announced on the website. We made this contest exclusive to medical students and decided on a participation fee of 5 Euros (US $5.79).

## Execution

On Saturday May 8, 2021, at 11 AM, the Zoom meeting was started by the president of Cerrahpaşa Neuroscience Society (MK) and the conference’s main moderator (ZO). The livestreams over YouTube and Twitch platforms started simultaneously, which caused the first technical issue of the conference as the streams were set to start later. The announced schedule had indicated 11:30 AM as the starting time; however, the mail sent to the participants stated that the Zoom meeting would be started at 11 AM, for the attendees who would like to use the Zoom platform. However, the Zoom membership only allowed 100 people for the meeting, and the waiting room was already full as our speaker was ready to enter. This resulted in the removal of many participants from the waiting room, and they were directed to the livestreams. At 11:50 AM, our team, our speaker, and the participants were ready to start as the livestream and the meeting problems were solved. Following opening speeches and brief remarks about attendance forms, the lectures were started.

During the first oral presentation session, minor technical difficulties were faced owing to a contestant not being familiar with the platform. However, this did not interfere with the flow of the program. No contestant used the given time slot completely; hence, an additional break was provided. The program then continued as planned.

On May 9, 2021, there was an update from the final speaker of the day: his session was to be interactive; therefore, our team needed to share a medical case with the participants and collect the answers to the related questions. For this purpose, a section on the organization website was designed with the aforementioned case and 3 related questions, and the answers were organized on a Google Sheets document. The moderator announced the questionnaire before the program for the day began. No other changes were made to the program and with the announcement of the contest winners, the 2021 Cerrahpaşa Neuroscience Days was over.

## Participants and Attendance

**Figure 1:**
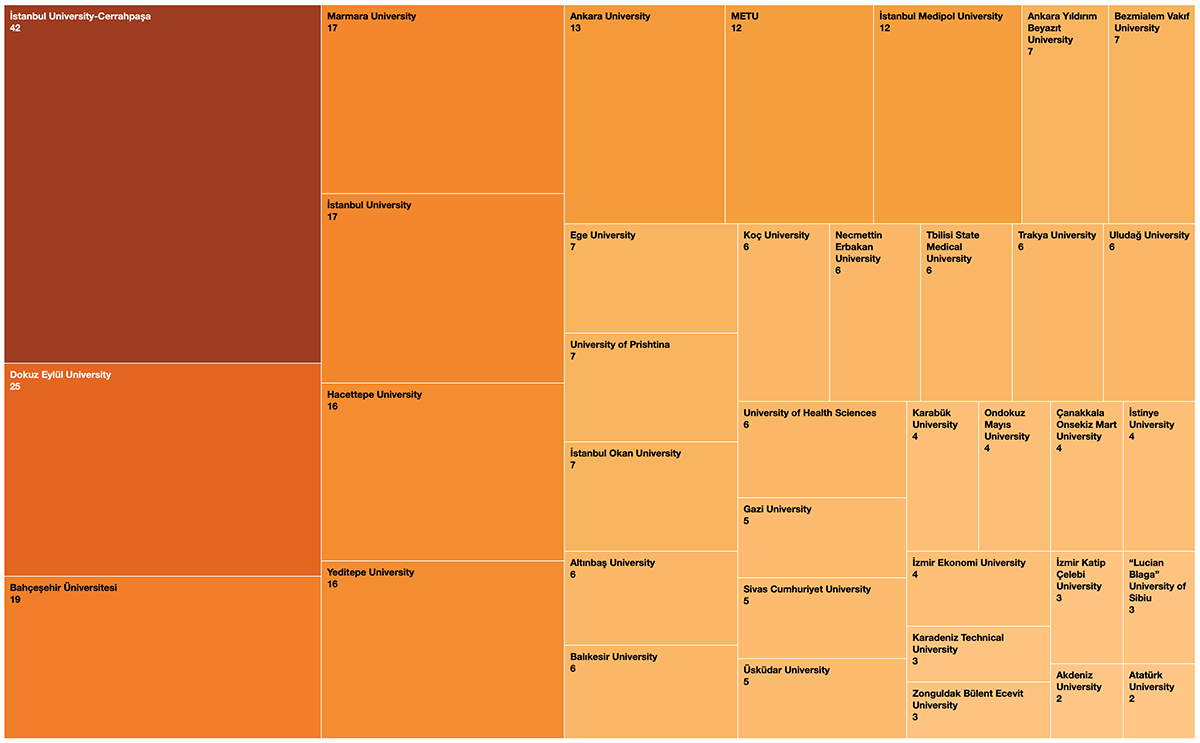
Participants' institutions.

**Figure 2:**
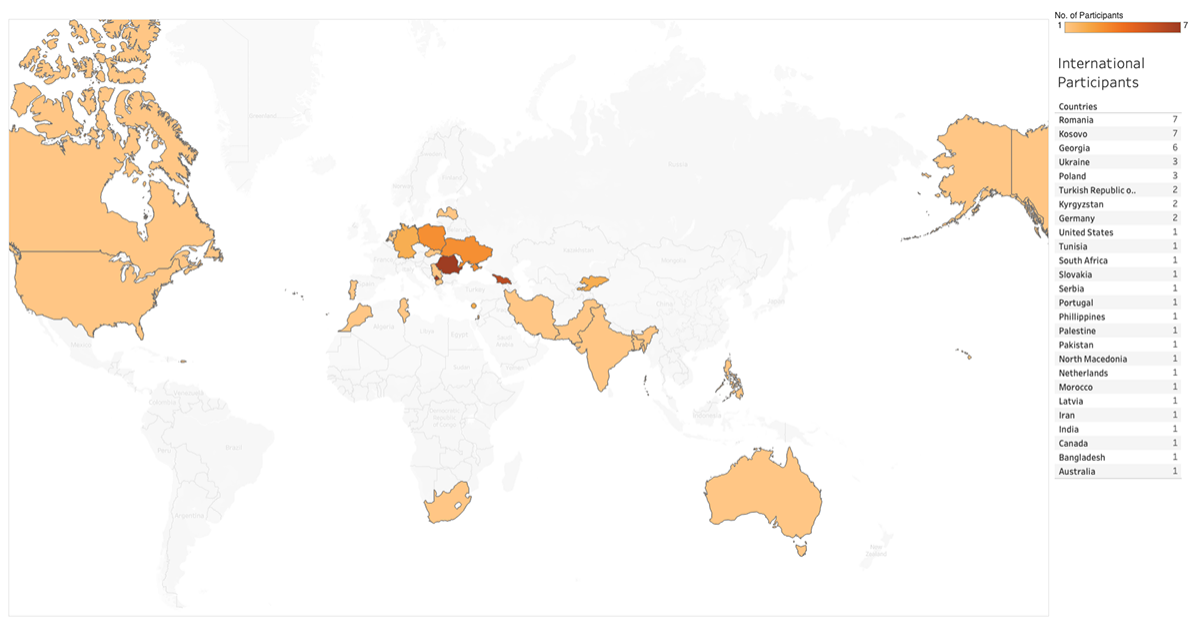
Participants' countries.

**Figure 3:**
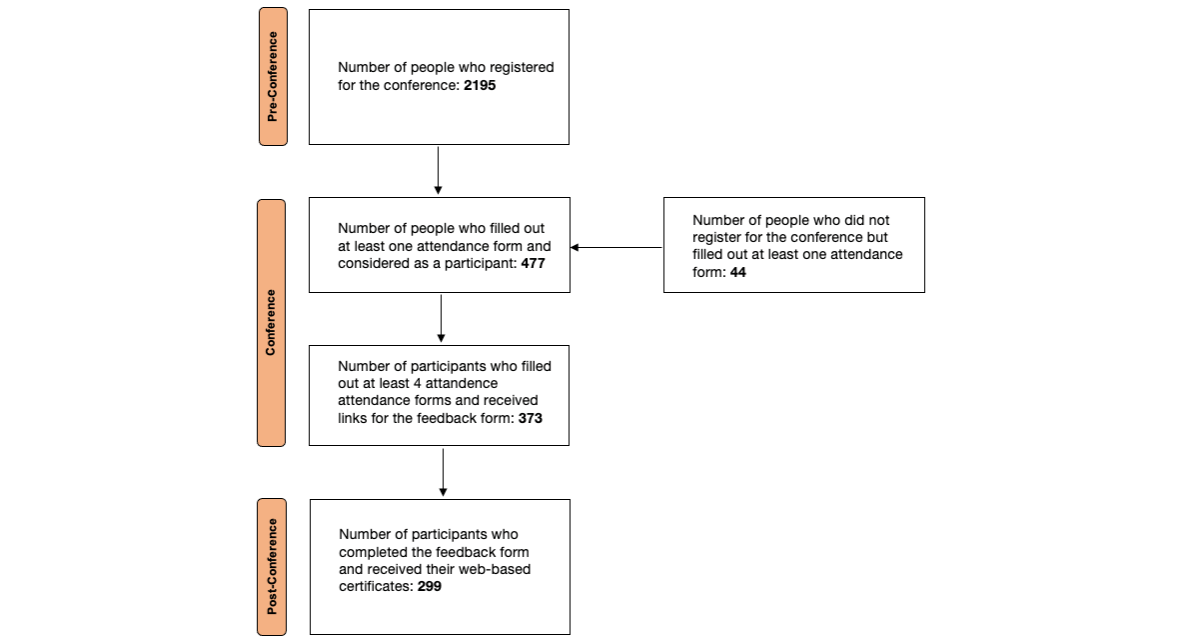
Registration and participation to the conference.

A total of 2195 people registered for the conference, 477 filled out at least 1 attendance form and were therefore considered participants, and 44 had not registered. The registered participants were from 115 different institutions in 27 different countries, with 18 people having registered as “independent,” not having provided the aforementioned information (Figure 1). The number of national and international registered participants was 365 and 50, respectively (Figure 2). Of the 477 participants, 373 filled out at least 4 out of 6 attendance forms and therefore received a feedback form link. In total, 299 of 373 filled out the feedback form and qualified for a certificate (Figure 3).

**Figure 4:**
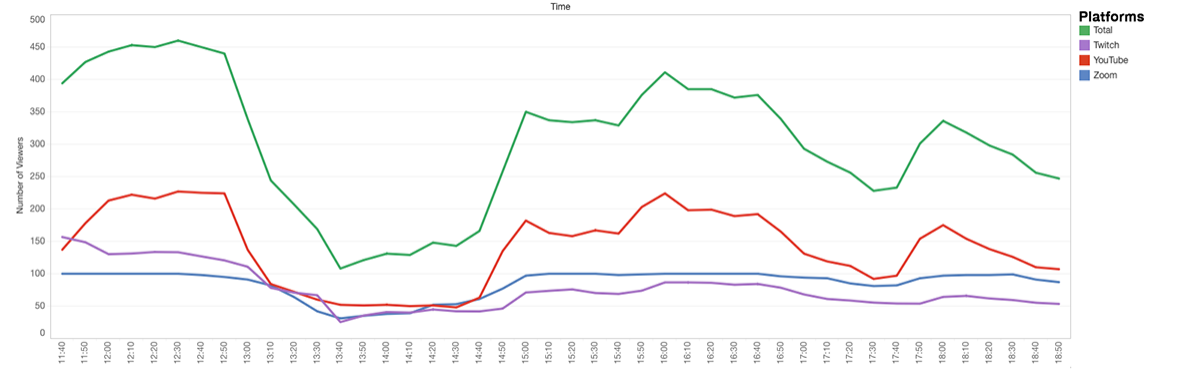
View counts on May 8 2021, Saturday.

**Figure 5:**
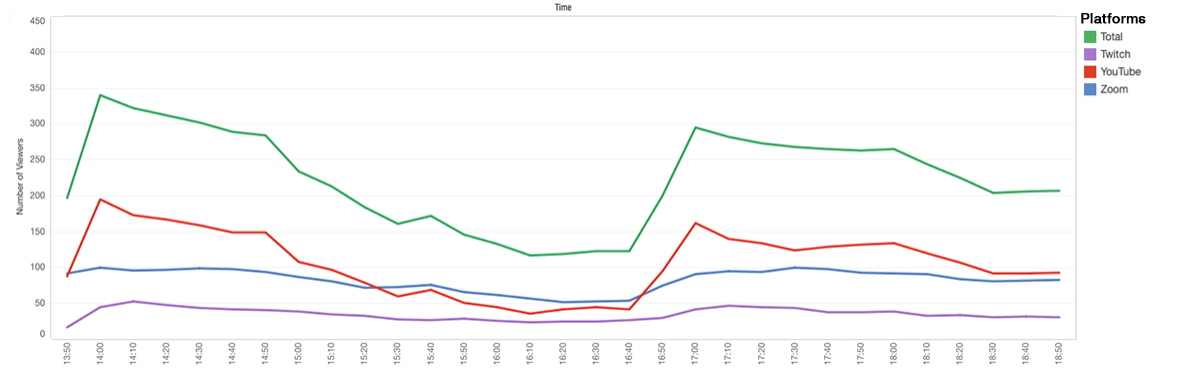
View counts on May 9 2021, Sunday.

The views from the start to the end of the conference, for 2 days, were analyzed. The peak view number was 460, reached during the first lecture. The second day of the conference had a peak view number of 340. The lowest view number on May 8 was 108 and 117 on May 9, both seen during break times (Figures 4 and 5).

## Feedback

A feedback questionnaire was prepared, and the e-portfolio platform MedAll was used to collect and categorize feedback. The questionnaire consisted of a total of 19 questions. In total, 14 of these questions were general evaluations regarding the event, based on a 5-point Likert scale with 1=“poor” and 5=“outstanding.” Through 3 open-ended questions, the participants were asked to list the strengths and weaknesses of the event, along with providing suggestions for future organizations. One question was designed to determine how the participants had learned about the event, and one question was regarding obtaining the medical or nonmedical background of the participants. To obtain a certificate, the participants who had filled at least 4 out of 6 participation forms were sent the feedback form and asked to fill it out. Names of the participants were not asked on the form. The responses of the participants were categorized to obtain an overview of how the event was perceived.

**Figure 6:**
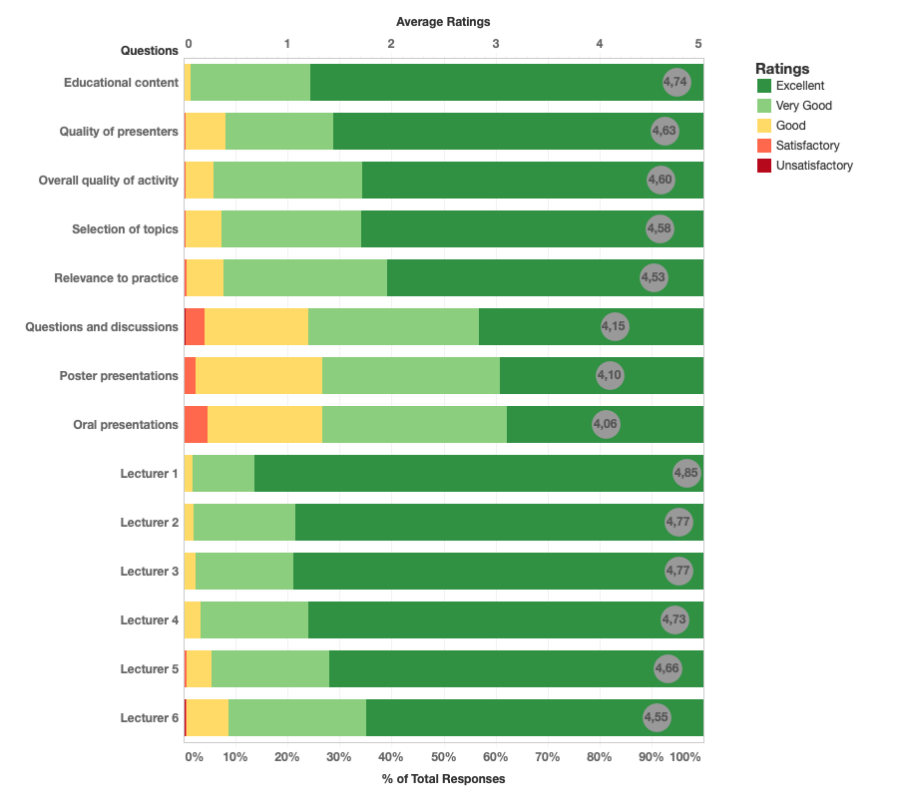
Results of feedback forms.

In total, 373 participants matched the criteria to fill out the feedback form. Out of these participants, 299 participants filled out the questionnaire and received their certificates on the internet. Lectures of the speakers were evaluated separately. Owing to all of the professors being renowned in their areas of expertise and because a student-based assessment is not adequate to reflect the proficiency of the speakers, we have decided not to disclose the individual feedback ratings of the lecturers in this paper. The contests were evaluated separately as well as overall aspects of the conference, such as the educational content and the selection of topics. These rating averages along with the scoring distribution are visualized in Figure 6.

Through open-ended questions, the organizers learned about the perceived strengths and weaknesses of the event, along with receiving suggestions on how to improve the conference in the future. Speaker choices were appreciated while the short duration of the question and answer sessions was pointed out as a weakness. For improvement, it was mostly asked that oral presentation sessions have a different approach.

In total, 143 participants had learned about the conference through social media. Twenty participants learned about the event over their email subscription, and 136 participants learned about the conference from their “friends/colleagues.”

Of the 299 participants, 221 had a medical education background, whereas 78 were considered as having a nonmedical background.

Statistical analysis was performed with SPSS Statistics (version 28). Cronbach α and corrected item-total correlations (CITCs) were used to assess the internal consistency of the scales in terms of reliability. Cronbach α was calculated and reached 0.883 (target value>0.7). CITCs ranged from 0.402 to 0.688 (target value>0.3).

## Discussion

Cerrahpaşa Neuroscience Days reached several students from many countries in many continents owing to various factors. With the internet-based format, any logistic issues were prevented, and students who would not be able to join a face-to-face conference could attend the event. We wanted as many students as possible to benefit from the lecturers of our conference; therefore, our conference was open-access. Moreover, with almost no financial requirements, we also decided to not set a registration fee. These served as encouraging factors for students who are eager to learn.

Despite the conference receiving over 2100 passive participation registrations, only 299 (~13%) participants complied with the certification criteria. The number of people who filled at least 1 participation form and were therefore considered participants was 477, with 44 not having registered previously. The numbers indicate crucial lessons about both a virtual format and the organization. The number of virtual events—in other words, easily accessible conferences—has skyrocketed during the pandemic. Even though this accessibility was and is still quite exciting, especially for students, it has also been overwhelming. It is quite easy to decide not to join the event as it is just one click away, and does not require planning ahead. Thus, the registrations and actual participation do not quite overlap. In addition to this factor, our conference was free of charge. This might have contributed to the excessive amount of registration, which was not realized.

When the feedback results were analyzed, an overall satisfaction among participants was observed**.** This is supported by the literature on web-based conferences: in the virtual German Rheumatology Congress, 80% of participants were either “satisfied” or “very satisfied” with the conference [1]. Similarly, II Dermachat Congress survey results showed that 61.6% of the attendees who had also attended the previous face-to-face format found the web-based version of the conference “superior” to its face-to-face counterpart [2]. The lectures of the speakers had the highest ratings. Similarly, feedback on the strengths of the conference mostly focused on how inspiring it was to listen to the professors. Along with the lectures being captivating, the speakers were from the most internationally acknowledged prestigious universities, and this reinforced the positive feedback in this aspect. The only common negative feedback on the lecture sessions was that the duration of the question-and-answer session was not long enough. Every speaker was given a total of 1 hour, including both the lecture and discussion. Our team did not have much control over how long the speeches would last; therefore, some speakers had more time left to answer questions, while some barely had time for one. A solution would be for the host to remind the professors of the duration limit before yielding the floor, so they can arrange their lectures.

The oral presentation sessions, in contrast, had the lowest rating. This was followed by the poster presentations. This correlates with the answers to the open-ended questions where the participants asked the presentation sessions to be handled differently. The majority of the problems regarding the presentations were in the oral presentation sessions and they were due to the lack of experience of the presenters with the platform. However, even when the presentation sessions proceeded as planned, there was a drastic decrease in the view numbers. This was expected, as the oral and poster presentations of the students were not the focus of the conference but rather a platform for students who wanted to gain experience in presenting in front of a large and diverse audience, as well as sharing their research with the attendees. The feedback reinforced this idea. Nonetheless, it would be inspiring to the participants and encouraging for the presenting students to have a larger active audience.

In terms of organization, as shown above, time management of our team was appreciated, except for the misunderstanding at the very beginning of the conference. Technical difficulties have been reported in the literature as in the 2020 Cochrane Skin Conference, sound transmission was not an uncommon issue with several reasons [9]. Nonetheless, these problems can be minimized, and the most important step in this process is to have a technical team working together and informing the participants about any encountered issue. The 2020 midterm conference of the Karnaka State Chapter of the Association of Surgeons in India reported having a technical team with members in different places with the prerecorded lectures in case of a network connectivity problem [10]. Such solutions could help resolve the issues that a web-based format could cause.

### Conclusions

As medical students, the increasing prevalence of web-based conferences has helped us introduce our society and events to an international audience. The virtual format also allowed our conference to be open-access and free of charge, which were encouraging factors for our audience. Students from various countries both presented and viewed the event, and the event received excellent feedback. As an undergraduate organization, these were pivotal for our society’s representation among the international community. Our team gained a great amount of experience from this organization with it being both virtual and completely student-based, and we hope that this paper helps as a guide for other organizations as they plan their conferences.

## Abbreviations

CITC: corrected item-total correlations
